# G-protein-coupled receptor kinase 2 terminates G-protein-coupled receptor function in steroid hormone 20-hydroxyecdysone signaling

**DOI:** 10.1038/srep29205

**Published:** 2016-07-14

**Authors:** Wen-Li Zhao, Di Wang, Chun-Yan Liu, Xiao-Fan Zhao

**Affiliations:** 1 Shandong Provincial Key Laboratory of Animal Cells and Developmental Biology, School of Life Sciences, Shandong University, Jinan, Shandong 250100, China

## Abstract

G-protein-coupled receptors (GPCRs) transmit extracellular signals across the cell membrane. GPCR kinases (GRKs) desensitize GPCR signals in the cell membrane. However, the role and mechanism of GRKs in the desensitization of steroid hormone signaling are unclear. In this study, we propose that GRK2 is phosphorylated by protein kinase C (PKC) in response to induction by the steroid hormone 20-hydroxyecdysone (20E), which determines its translocation to the cell membrane of the lepidopteran *Helicoverpa armigera*. GRK2 protein expression is increased during the metamorphic stage because of induction by 20E. Knockdown of *GRK2* in larvae causes accelerated pupation, an increase in 20E-response gene expression, and advanced apoptosis and metamorphosis. 20E induces translocation of GRK2 from the cytoplasm to the cell membrane via steroid hormone ecdysone-responsive GPCR (ErGPCR-2). GRK2 is phosphorylated by PKC on serine 680 after induction by 20E, which leads to the translocation of GRK2 to the cell membrane. GRK2 interacts with ErGPCR-2. These data indicate that GRK2 terminates the ErGPCR-2 function in 20E signaling in the cell membrane by a negative feedback mechanism.

G-protein-coupled receptors (GPCRs) are important cell membrane proteins that transmit environmental and physiological signals, including light, neurotransmitters, odorants, gustatory substances, and hormones, into the cells^1^. Termination of a GPCR signal is regulated by GPCR kinases (GRKs) and arrestin^2^. GRKs phosphorylate the active GPCRs to convert GPCRs into a target for arrestin binding. Arrestin interacts with phosphorylated GPCRs to internalize GPCR^3^ or to desensitize GPCR signals by combining with GPCR^4^ to prevent GPCR coupling to G proteins for signaling.

GPCRs transmit steroid hormone signal in the cell membrane such as GPR30, which binds estrogen and triggers the rapid cell responses^5^. GPCRs also transmit 20-hydroxyecdysone (20E) signal to induce rapid cellular calcium increase in the silk glands of *Bombyx mori*^6^. In *Helicoverpa armigera*, the ecdysone-responsive GPCR1 (ErGPCR-1) transmits 20E signals in the cell membrane, including rapid cellular calcium increase, protein phosphorylation, and subcellular translocation, which regulate the formation of ecdysone nuclear receptor/ultraspiracle protein (EcR/USP) transcription complex, gene transcription, and metamorphosis^7^. Another GPCR, ErGPCR-2, transmits 20E signals from the cell membrane to the nucleus for gene expression^8^. ErGPCR-2 localizes in the cell membrane and controls the entrance of 20E analog [^3^H]Pon A into the cells. GRK2 directly phosphorylates ErGPCR-2 and induces the internalization of ErGPCR-2, thereby decreasing the entrance of 20E and terminating the 20E signal^8^. However, the mechanism of GRK2 activation in termination of steroid hormone signals remains unclear.

The membrane localization of GRKs is crucial for their functions and the C-terminal of GRKs is critical for their membrane localization^9^. The CaaX motif at C-terminal of GRKs 1 and 7 are isoprenylated (farnesylated), which brings them to the membrane under light exposure to shut off the photoreceptor response to light^10^. The pleckstrin homology (PH) domain at the C-terminal of GRKs 2 and 3 interacts with G protein βγ (Gβγ)^11^, and the odorant-stimulated translocation of GRK3 from cytosol to the membranes is Gβγ binding-dependent in mice^12^. The C-terminal of GRKs 4 and 6 are palmitoylated under adrenaline stimulation to terminate the β2AR response to adrenaline^13^,^14^. Phosphorylation is another mechanism of GRK membrane localization. In HEK293 cells, GRK2 responds to isoproterenol and is phosphorylated by protein kinase A (PKA), and then translocated to the cell membrane^15^. PKC phosphorylation of GRK2 at C-terminal increases its catalytic activity towards the beta-adrenergic receptor^16^ and rhodopsin^17^. GRK2 phosphorylates muscarinic acetylcholine receptor (mAChR) to terminate the carbachol signal in human tsA201 cells^18^. GRK2 and GRK3 participate in the rapid termination of the odorant receptors (ORs) by phosphorylation of ORs^19^,^20^. GRK4 is implicated in the regulation of blood pressure and in the autophosphorylation of GRK4 at serine 139 and serine 485^9^.

We investigated the phosphorylation of GRK2 in response to induction by the steroid hormone 20E to determine the mechanism of GRK2 translocation into the cell membrane to terminate 20E signal. Results showed that 20E induced the increase in GRK2 expression during metamorphosis. 20E regulates the phosphorylation of GRK2 at serine 680, which causes the translocation of GRK2 to the cell membrane. GRK2 interacts with ErGPCR-2 and terminates ErGPCR-2 function in 20E signaling in the cell membrane via a negative feedback mechanism.

## Results

### GRK2 is highly expressed during metamorphosis

**Figure 1:**
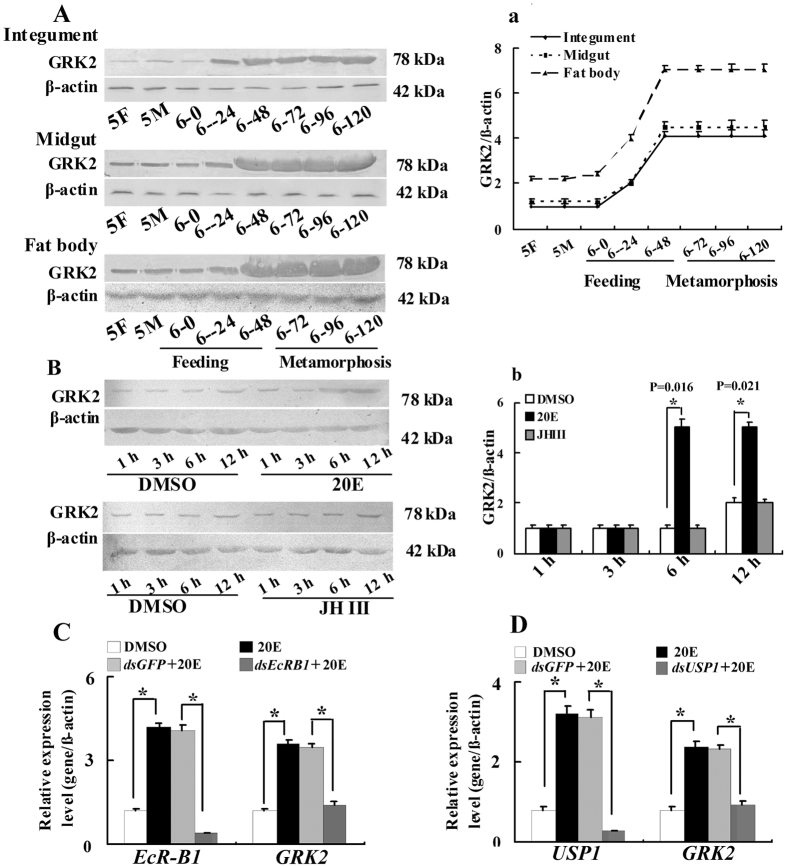
Western blot analysis showing GRK2 expression profiles during larval development. (**A)** The GRK2 expression levels in the integument, midgut and fat body detected using an antibody against *H. armigera* GRK2. β-actin was used as the control and was detected using an antibody against *H. armigera* β-actin. **5F:** fifth instar feeding larvae 24 h after ecdysis; **5M:** fifth instar molting larvae; 6-0, 6-24, 6-48, 6-72, 6-96, and 6-120 represent sixth instar larvae at the corresponding times. Figure S5A are the full-length blots data **a**. Quantitative analysis of (**A**) using ImageJ software. (**B**) The sixth instar 6 h larvae were injected with 20E or JH III (500 ng/larva) for 1, 3, 6 and 12 h, and the integument proteins were examined (30 larvae, three triplicates). The sixth instar 6 h larvae were injected with equivalent volume of DMSO for 1, 3, 6 and 12 h as the control group (30 larvae, three triplicates). β-actin was used as the control. Figure S5B are the full-length blots data **b**. Statistical analysis of (**B)** according to the quantification of the bands with ImageJ software. Asterisks indicate significant differences between the groups (p < 0.05) by the Student’s t-test based on three independent experiments. The bars indicate the means + SD of three independent experiments. (**C,D**) 20E via EcRB1 and USP1 regulates *GRK2* expression by qRT-PCR analysis. The cells were treated with *dsEcRB1* or *dsUSP1* (1 μg/ml for 12 h) and/or 20E (1 μM for 6 h). *P < 0.05 (Student’s t-test), based on three independent experiments. Error bars indicate the means + SD of three independent biological experiments

To study the function of GRK2 in the 20E pathway, we examined the expression profiles of GRK2 protein in the epidermis, midgut, and fat bodies from the fifth instar to the prepupae and the 20E-dependent regulation of GRK2 expression. GRK2 was expressed at a lower level from the fifth instar stage to the sixth instar at 0 h. However, GRK2 expression markedly increased from the sixth instar at 24 h, and then maintained a stable high expression level to the prepupal stage (Fig. 1A). This expression pattern suggests that GRK2 expression is regulated by 20E during metamorphosis because the 20E titer is higher during molting and metamorphosis in lepidopterans^21^. Therefore, we injected sixth instar 6 h larvae with juvenile hormone III (JH III) or 20E to evaluate the hormone’s regulation of GRK2 expression. The results showed that 20E upregulated the expression of GRK2 in the integument, however, JH III did not upregulate the expression of GRK2 (Fig. 1B). 20E up regulated the expression of *GRK2* via EcR-B1 and USP1 (Fig. 1C,D). These results suggested that GRK2 expression increases during metamorphosis and is upregulated by 20E.

### Knockdown of *GRK2* promoted metamorphosis and gene expression in the 20E pathway

**Figure 2:**
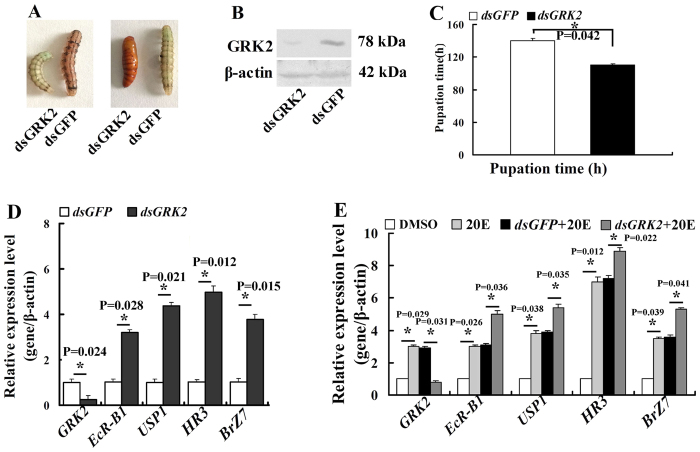
GRK2 suppressed 20E-induced metamorphosis and gene expression. (**A)** Phenotypes 60 h after *GRK2* knockdown (injection of *dsGRK2* into sixth instar 6 h larvae, 500 ng/larva, twice in a 48-h interval) with *dsGFP* as the control. (**B**) Western blot analysis showing the efficacy of *GRK2* knockdown in integument after 60 h from tissue homogenate of three larvae. β-actin was used as the control. Figure S6 are the full-length blots data. (**C)** Statistical analysis of the pupation time of 50% larvae (*P*_50_) after *GRK2* knockdown (30 larvae, three triplicates) by the Student’s t-test. Asterisks indicate significant differences between the groups (p < 0.05) by the Student’s t-test. (**D**) Gene expression in the integument after *GRK2* knockdown in larvae (500 ng of *dsGRK2*/larva, extracted RNA 48 h after first injection). Asterisks indicate significant differences between the groups (p < 0.05) by the Student’s t-test based on three independent experiments. (**E**) qRT-PCR analyses showing gene expression in HaEpi cells after *GRK2* knockdown (1 μg/mL of *dsGRK2*, 1 μM 20E for 12 h). Asterisks indicate significant differences between the groups (p < 0.05) by the Student’s t-test based on three independent experiments. The bars indicate the means + SD of three independent experiments.

We knocked down *GRK2* by injecting *dsGRK2* into the sixth instar 6 h larvae to examine the function of GRK2 in the 20E pathway. *GRK2* knockdown accelerated pupation compared with the *dsGFP*-injected control larvae. The pupation was accelerated after *dsGRK2* injection compared with *dsGFP*-treated larvae (Fig. 2A). The efficacy of *GRK2* knockdown was confirmed by Western blot using integument homogenates (Fig. 2B). The pupation time from sixth instar 0 h to pupa in *dsGRK2*-injected larvae was 110 h, which was 30 h earlier than the 140 h in *dsGFP*-injected control larvae (Fig. 2C). These results suggest that GRK2 functions prevent 20E-induced metamorphosis.

To address the mechanism of *GRK2* knockdown that causes accelerated metamorphosis, we examined the expression profile of a series of genes in larval integument after *GRK2* knockdown, including the 20E pathway genes *EcR-B1*, *USP1*, *Br-Z7*, and *HR3*, using qRT-PCR. *EcR-B1*, *USP1*, *Br-Z7*, and *HR3* expression levels were significantly upregulated after *GRK2* knockdown compared with the *dsGFP* control (Fig. 2D). *GRK2* expression was knocked down in *H. armigera* epidermal cells (HaEpi) to exclude the differences of the developmental stages from *dsGRK2 and dsGFP*-treated larvae. Results showed that knockdown of *GRK2* also increased 20E-induced genes (*EcR-B1*, *USP1*, *HR3*, and *BrZ7*) expression levels (Fig. 2E). These results suggested that GRK2 represses 20E-pathway gene expression.

### Knockdown of *GRK2* promoted 20E-induced apoptosis

**Figure 3:**
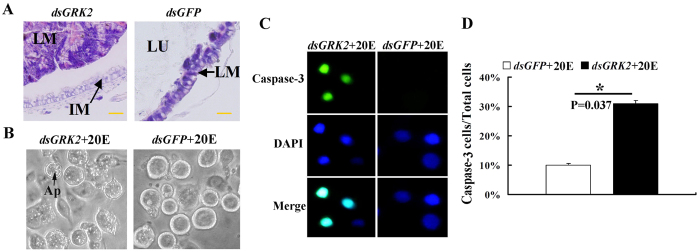
GRK2 suppressed 20E-induced apoptosis. (**A)** Hematoxylin and eosin (HE) staining showing midgut remodeling 60 h after *dsGRK2* injection (500 ng dsRNA/larva). **Lu**, midgut lumen; **LM**, larval midgut; **IM**, imaginal midgut. The bar represents 20 μm. (**B**) The cellular morphology (observed by Nikon eclipse TS100 microscopy) in *GRK2* knockdown cells after incubation with 20E for 72 h (1 μg of *dsGRK2* for 24 h and then 1 μM 20E for 72 h); *dsGFP* + 20E is the control. Ap: apoptotic vesicles. (**C**) Caspase-3 activity in *GRK2* knockdown cells (1 μg *dsGRK2*, 1 μM 20E for 72 h); *dsGFP* + 20E is the control. Green fluorescence indicates Caspase-3 activity. Blue indicates nuclear stained by 4-6-diamidino-2-phenylindole dihydrochloride (DAPI). Merge is the overlapped green and blue. (**D**) Quantification of caspase-3-stained cells in (**C**) was performed using Image pro plus. The bars indicate the means + SD of three independent experiments.

Given that midgut apoptosis is a typical characteristic of metamorphosis by 20E regulation^22^, we observed the occurrence of apoptosis in the midgut and HaEpi cells after *GRK2* knockdown because RNA interference (RNAi) is generally efficient in lepidopteran larvae^23^. Sixty hours after *dsGRK2* injection, the midgut displayed characteristics of apoptosis, including condensation of the larval midgut toward the midgut lumen and the separation of the larval midgut from the newly formed imaginal midgut. By contrast, 60 h after *dsGFP* injection, the midgut maintained feeding characteristics, without condensation of the larval midgut, imaginal midgut formation, and separation of the larval midgut from imaginal midgut (Fig. 3A). These results suggested that GRK2 functions to prevent midgut apoptosis.

We knocked down *GRK2*, followed by 1 μM 20E treatment in HaEpi cells to exclude the differences of the developmental stages from *dsGRK2 and dsGFP*-treated larvae and to confirm the apoptosis by *GRK2* knockdown. The *GRK2* knockdown increased 20E-induced apoptotic vesicles in the cells under 1 μM 20E induction at 72 h (Fig. 3B). The caspase-3 activity was further detected to confirm the apoptosis by *GRK2* knockdown. The *dsGRK2*-treated cells presented a significant increase of the caspase-3 activity under 1 μM 20E induction at 72 h, however, the *dsGFP*-treated control cells presented a lesser caspase-3 activity under 1 μM 20E induction at 72 h (Fig. 3C,D). These results suggested that *GRK2* represses 20E-induced apoptosis.

### GRK2 moves toward the cell membrane in 20E induction in HaEpi cells

**Figure 4:**
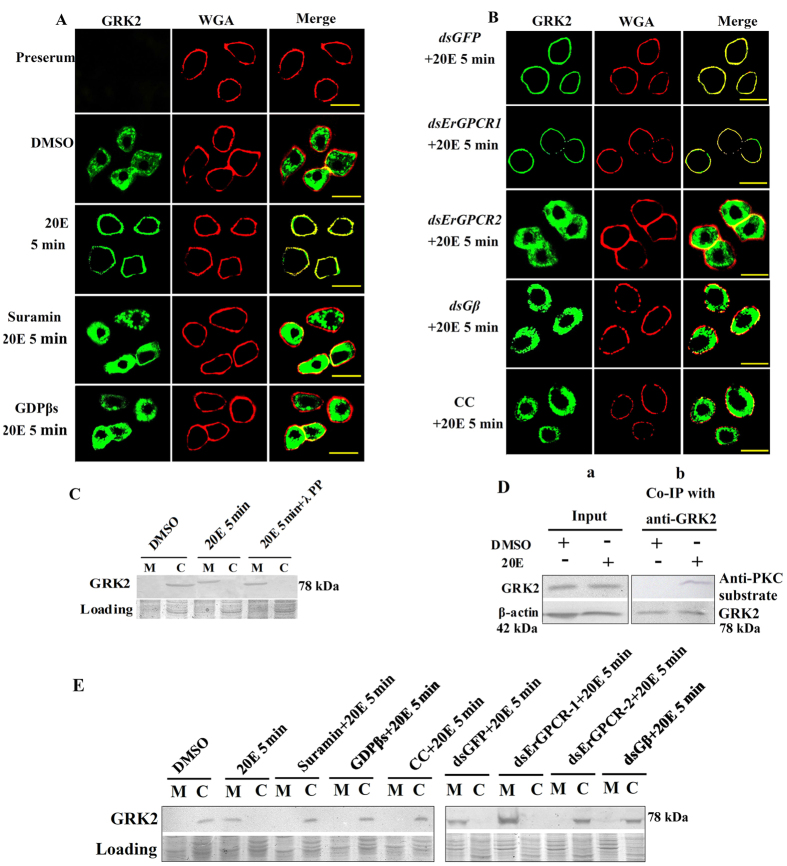
GRK2 relocates toward the cell membrane after 20E induction. (**A**,**B**) Immunocytochemical analysis of GRK2 (green color) after different treatments using a Zeiss LSM 700 laser confocal microscope: 20E (1 μM for 5 min), suramin (50 μM for 60 min) + 20E (1 μM for 5 min), GDPβs (100 μg/mL) + 20E (1 μM for 5 min), *dsErGPCR-1* (1 μg/mL), + 20E (1 μM for 5 min), *dsErGPCR-2* (1 μg/mL) + 20E (1 μM for 5 min), CC (5 μM for 60 min) + 20E (1 μM for 5 min) by an antibody against *H. armigera* GRK2. The control received an equivalent volume of DMSO. Then 1 μg/mL Alexa Fluor 594-conjugated wheat germ agglutinin (WGA) was incubated with the cells in DPBS for 15 min at room temperature to label the plasma membrane (red color). Merge is the overlapped green and red. The yellow bar denotes 20 μm. **(C**) Western blots to confirm the phosphorylation of GRK2 after 20E treatment using anti-*H. armigera* GRK2. SDS-PAGE gel with Coomassie Brilliant Blue staining was performed at the same time as protein loading control to normalize the protein amounts in the membrane (M) and cytoplasm (C). λ PP: λ-protein-phosphatase (5 mM, 30 min at 30 °C). Figure S7 are the full-length blots and gels data. The gels ran under the same experimental conditions. (**D**) Analysis of the PKC phosphorylation of GRK2 (20E 1 μM for 5 min). **Input:** protein expression levels of GRK2 and β-actin in various treated cells using antibodies against *H. armigera* GRK2 and β-actin. β-actin was used as the loading control. Co-IP with anti-GRK2 and then detection with anti- phospho-PKC substrate antibody. The PKC substrate antibody was a polyclonal antibody against the phospho-(Ser) PKC substrate antibody which from Cell Signaling Technology Inc, USA. Figure S8 are the full-length blots data. (**E**) Western blotting to confirm the subcellular distribution of GRK2 after different treatments that were the same as in (**A**,**B**) using an antibody against *H. armigera* GRK2. SDS-PAGE gel with Coomassie Brilliant Blue staining was performed at the same time as loading of the control to normalize the protein amounts in the membrane (M) and cytoplasm (C). Figure S9 are the full-length blots and gels data. The gels ran under the same experimental conditions.

We examined the hormonal regulation of GRK2 subcellular localization in HaEpi cells within 5 min after 20E induction to determine the mechanism of GRK2 function in 20E signaling. GRK2 was localized in the cytoplasm of the dimethylsulfoxide (DMSO) treatment control. However, GRK2 localized to the cell membrane under 20E induction. Suramin (a broad-spectrum antagonist of P2 receptors^24^,^25^, agonist of Ryanodine receptors^26^ and inhibitor of GPCRs)^27^ and GDPβs (the GPCR inhibitors) repressed the 20E-induced membrane localization (Fig. 4A), suggesting that 20E induced GRK2 membrane translocation from the cytoplasm via the GPCRs. Because these experiments were performed by 5 min 20E induction, there was no obvious variation on GRK2 expression levels.

We knocked down *ErGPCR-1* that transmits 20E signal^7^ and *ErGPCR-2* that controls 20E entrance to determine which GPCR participated in 20E-induced GRK2 membrane translocation^8^. *ErGPCR-1* knockdown had no effect on the 20E-induced GRK2 membrane localization; however, *ErGPCR-2* knockdown repressed the 20E-induced GRK2 membrane localization. *Gβ2* knockdown and the PKC inhibitor chelerythrine chloride (CC)^28^ also repressed the 20E-induced GRK2 membrane localization (Fig. 4B). These data suggest that 20E regulated GRK2 membrane localization via ErGPCR-2 and that Gβ2 and PKC are involved in 20E-induced GRK2 membrane localization.

Western blotting was performed to confirm the membrane localization of GRK2 under 20E induction. Results showed that 20E induced membrane localization of GRK2 in 5 min. The molecular mass of the membrane-located GRK2 was decreased by λ-protein-phosphatase (Fig. 4C), indicating the phosphorylation of GRK2. GRK2 was proven to be phosphorylated in 5 min in response to 20E induction by anti-PKC substrate antibody detection (Fig. 4D). The results of western blotting also agreed with the above immunocytochemistry results that 20E induced GRK2 membrane localization. However, suramin, GDPβs, CC, or knockdown of *ErGPCR-2* and *Gβ2* repressed 20E-induced GRK2 membrane localization (Fig. 4E). These results suggested that 20E regulates GRK2 membrane translocation and PKC phosphorylation.

### Phosphorylation of serine 680 determines the membrane localization of *GRK2*

**Figure 5:**
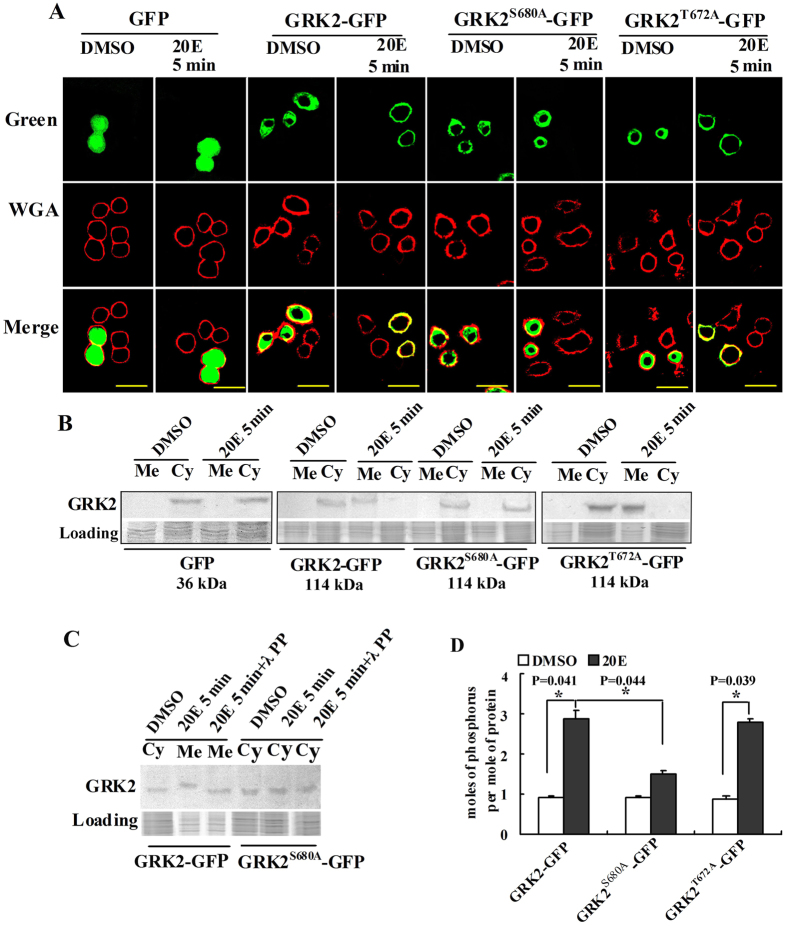
Serine 680 phosphorylation determines GRK2 membrane migration in response to induction by 20E. (**A**) Green fluorescent protein (GFP), GRK2-GFP, GRK2^T672A^-GFP-His and GRK2^S680A^-GFP-His (green) were overexpressed by the plasmid GFP-pIEx-His in HaEpi cells after treatment with 20E (1 μM) or an equal volume of the DMSO control for 5 min and were viewed using a Zeiss LSM 700 laser confocal microscope. WGA (red) was used as a cell membrane marker. Merge is the overlapped green and red. The yellow bars denote 20 μm. (**B**) Western blot analysis for the overexpression and subcellular distribution of GRK2-GFP-His, GRK2^T672A^-GFP-His (114 kDa) and GRK2^S680A^-GFP-His (114 kDa) in HaEpi cells, which were detected using an antibody against GFP-tag. Cytoplasm (Cy) and membrane (Me) proteins were extracted after treatment with 20E (1 μM) or an equal volume of DMSO as a control for 5 min. SDS-PAGE with Coomassie Brilliant Blue staining was performed at the same time as loading of controls to normalize the protein amounts in the membrane and cytoplasm. Figure S10 are the full-length blots and gels data. The gels ran under the same experimental conditions. (**C**) Western blots to confirm phosphorylation of GRK2-GFP-His after 20E treatment by contrast with GRK2^S680A^-GFP-His using anti-GFP. SDS-PAGE gel with Coomassie Brilliant Blue staining was performed at the same time as loading of the control to normalize the protein amounts in the membrane (Me) and cytoplasm (Cy). λ PP: λ-protein-phosphatase (5 mM, 30 min at 30 °C). Figure S11 are the full-length blots and gels data. The gels ran under the same experimental conditions. (**D**) Numbers of moles of phosphorus per mole of GRK2-GFP-His, GRK2^T672A^-GFP-His and GRK2^S680A^-GFP-His were analyzed using a phosphoprotein phosphate estimation kit. The 20E concentration was 1 μM for 5 min. DMSO was used as the control. Asterisks indicate significant differences between the groups (p < 0.05) that were determined by Student’s t-test based on three independent experiments. The bars indicate the means + SD of three independent experiments.

The PKC phosphorylation site in GRK2 was identified by site mutation to address the relationship between PKC phosphorylation and GRK2 membrane localization. There were two ser/thr PKC phosphorylation sites in GRK2 at threonine 672 and serine 680 that were predicted by NetPhosK 2.0 analysis (http://www.cbs.dtu.dk/services/NetPhos/). Wild-type GRK2 (GRK2-GFP-His) and site mutants of GRK2 obtained by replacing threonine with alanine (GRK2^T672A^-GFP-His) or by replacing serine with alanine (GRK2^S680A^-GFP-His) were overexpressed in HaEpi cells. GFP-His overexpression was used as the nonspecific protein control. The green fluorescent signal was detected in the entire cell in the GFP-His-overexpressing cells. The green fluorescent signals were detected in the cytoplasm in of cells that express GRK2-GFP-His, GRK2^T672A^-GFP, and GRK2^S680A^-GFP. GRK2-GFP-His and GRK2^T672A^-GFP were transferred to the membrane within 5 min after induction by 20E. However, GRK2^S680A^-GFP-His was unable to move toward the cell membrane within 5 min after induction by 20E (Fig. 5A). These results suggest that serine 680 phosphorylation determined the membrane localization of GRK2. These results were confirmed by western blot analysis (Fig. 5B). The molecular mass of the membrane-located GRK2-GFP-His from 20E-treated cells was higher than that from DMSO control cells, which was decreased by λ-protein phosphatase, suggesting 20E induced GRK2 phosphorylation. By contrast, the molecular mass of GRK2^S680A^-GFP-His was not changed by 20E induction (Fig. 5C). The level of 20E-induced GRK2 phosphorylation was further examined using a phosphoprotein phosphate estimation assay kit. The average number of phosphates was detected as 3 per molecule of GRK2-GFP-His protein after induction by 20E. When threonine 672 was mutated, the phosphorylation level of GRK2^T672A^-GFP-His was unchanged after induction by 20E, in contrast with the DMSO control. When serine 680 was mutated, the phosphorylation level of GRK2^S680A^-GFP-His was decreased because of induction by 20E (Fig. 5D), suggesting that 20E-induced phosphorylation occurred at serine 680 of GRK2. These data suggested that phosphorylation of GRK2 at serine 680 determines the migration of GRK2 toward the cell membrane.

### GRK2 combined with ErGPCR-2 under 20E treatment

**Figure 6:**
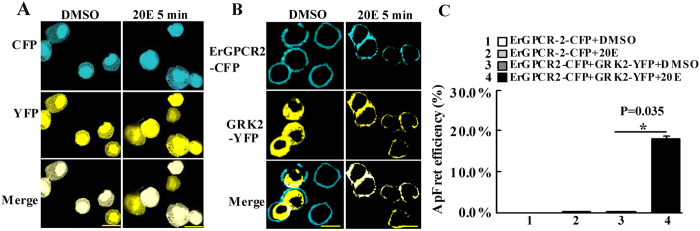
GRK2 interacts with ErGPCR-2. (**A)** CFP-pIEx-His (blue) and YFP-pIEx-His (yellow) plasmids were co-transfected in cells as controls. (**B**) ErGPCR2-CFP-pIEx-His and GRK2-YFP -pIEx-His plasmids were co-transfected in the cells. Cells were treated with DMSO or 20E (1 μM) for 5 min. Merge is the overlapped yellow and blue. The yellow bar denotes 20 μm. (**C**) Ap-Fret detected the interaction between ErGPCR-2 and GRK2. ErGPCR-2-CFP and GRK2-YFP plasmids were co-tranfected in cells. The overexpression of ErGPCR-2-CFP was the negative control. Cells were incubated with DMSO or 20E (1 μM for 5 min). Ap-Fret efficiency (%) = (Ipost − Ipre) × 100/Ipost, the Ap-Fret efficiency of ErGPCR-2-CFP overexpression as the negative control. Asterisks indicate significant differences between the groups (p < 0.05) by the student’s t-test based on three independent experiments.

Previous work indicated that GRK2 interacted with ErGPCR-2 in the cell membrane^8^. To confirm this finding, the co-localization of ErGPCR-2-CFP and GRK2-YFP and Ap-FRET experiments were further performed. The overexpressed ErGPCR-2-CFP and GRK2-YFP co-localized in the cell membrane 5 min after 20E induction (Fig. 6A,B). In Ap-FRET experiments, a significant increase of the FRET was observed after induction with 20E when ErGPCR-2-CFP and GRK2-YFP were co-transfected in the cells, in contrast to the DMSO control or ErGPCR-2-CFP (donor) by induction with DMSO or 20E (Fig. 6C). These data confirmed the interaction between GRK2 and ErGPCR-2.

## Discussion

GRKs desensitized GPCR signals depending upon their localization in the cell membrane. However, the mechanism by which translocation from the cytoplasm to the cell membrane of GRK2 in the induction of steroid hormone is unclear. This study revealed that steroid hormone 20E-induced membrane translocation of GRK2 from the cytoplasm is determined by PKC-mediated phosphorylation at serine 680 of GRK2. 20E regulated GRK2 PKC phosphorylation and translocation from the cytoplasm to the cell membrane via ErGPCR-2. GRK2 combined with ErGPCR-2 and phosphorylated ErGPCR-2 to induce ErGPCR-2 internalization and terminate ErGPCR-2 function in 20E signaling.

The GRK expression level provides the basis for its function in terminating GPCR signals. Increased expression levels of GRKs have been observed in various signal pathways. For example, estrogen increases GRK2 expression in the female *rat* cortex^29^,^30^. Glucocorticoids upregulate GRK2 expression in Brown-Norway Rat lungs^31^. Two peaks of 20E titer are found in the penultimate instar and last instar late stages in lepidoptera *Manduca sexta*^32^, *H. armigera*^21^, and *Antheraea mylitta*^33^. In the current study, we demonstrated that the steroid hormone 20E upregulated GRK2 expression via EcRB1 and USP1, and that the increased levels of GRK2 led to the phosphorylation and internalization of ErGPCR-2 because of 20E induction. The internalization of ErGPCR-2 resulted in the reduction of the steroid hormone signal because ErGPCR-2 is necessary for the entrance of the 20E analog [^3^H]Pon A into the cells^8^. This hypothesis was supported by the observations that 20E pathway genes were upregulated and precocious pupation was promoted after knockdown of *GRK2*.

GRK2 translocation from the cytoplasm to the cell membrane in response to extracellular signals is critical to the function of the protein, where GRK phosphorylation is an important component of its translocation to the membrane. GRK and its phosphorylation in mammals have been widely investigated. GRK5 is regulated by autophosphorylation in the presence of crude soybean phosphatidylcholine liposomes at its C-tail region at Ser484 and Thr485^34^. The phosphorylation of GRK2 by PKC or PKA is essential for the membrane recruitment of GRK2 in the induction of adrenaline or isoproterenol^15^,^16^. The phosphorylation of GRK2 by ERK1/2 in the induction of adrenaline reduces the GRK2 activity^35^. GRK2 is phosphorylated by PKA at serine 685 as a result of induction by isoproterenol in HEK293 cells, which promotes its interaction with Gβγ and membrane recruitment^15^. In COS-1 cells, GRK2 is phosphorylated by c-SC at Tyr 13, 86, and 92 after induction by isoproterenol, which enhances its activity towards GPCR^36^. In our study in *H. armigera*, GRK2 was phosphorylated at serine 680 via PKC, which determined its translocation to the cell membrane after induction by steroid hormone 20E.

The interaction between the PH domain of GRK2/3 and Gβγ is a classical mechanism for anchoring of GRK2/3 in cell membranes^11^,^37^. In mice, lipopolysaccharide, through p38-MAPK signaling, induces GRK2 phosphorylation at serine 670, which lies within the Gβγ binding domain, and suppresses GRK2 translocation to the membrane because MAPK phosphorylation at this site impairs its interaction with GRK2-Gβγ^38^. In addition, Gβ2 is essential for the membrane localization of GRK2. Therefore, GRK2 could be localized in the cell membrane by binding with Gβγ. Our study is the first to reveal the mechanism for GRK2 cell membrane translocation because of induction by steroid hormones.

The negative feedback control of signaling exists widely in various GPCRs^39^,^40^,^41^. The major molecules that are involved in GPCR signal desensitization are GRKs^42^. GRKs phosphorylate the ligand-activated receptors and promote high-affinity binding of arrestin with the GPCRs, which precludes G protein coupling^43^. In our previous work, we found that the 20E analog [^3^H]Pon A enters into the cells via ErGPCR-2. GRK2 phosphorylated ErGPCR-2 and resulted in the internalization of ErGPCR-2, which decreased the entrance of [^3^H]Pon A, thus terminating 20E signaling^8^. In the current work, we further demonstrated that 20E regulated PKC phosphorylation of GRK2 at serine 680 via ErGPCR-2, and the phosphorylated GRK2 translocated to the cell membrane, where it interacted with ErGPCR-2. In addition, the results of *GRK2* knockdown experiments suggest that GRK2 is a repressor of the 20E pathway. Considering the previous work^8^, this study presents an example of a negative feedback regulation mechanism between ErGPCR-2 and GRK2 because of regulation by the steroid hormone 20E. The 20E level is elevated in the metamorphic stage^33^, thus, the negative feedback control of 20E signaling by GRK2 might ensure sufficient development. The negative feedback control of 20E signaling by β-arrestin was also reported in a previous work^4^. Although ErGPCR-2 function is terminated by GRK2-mediated internalization, ErGPCR-2 is consistently upregulated by 20E^8^, which may compensate the shortage of ErGPCR-2 in the cell membrane, thus maintaining gene expression at an appropriate level. 20E finally regulates metamorphosis by this homeostasis of gene expression.

## Conclusion

**Figure 7:**
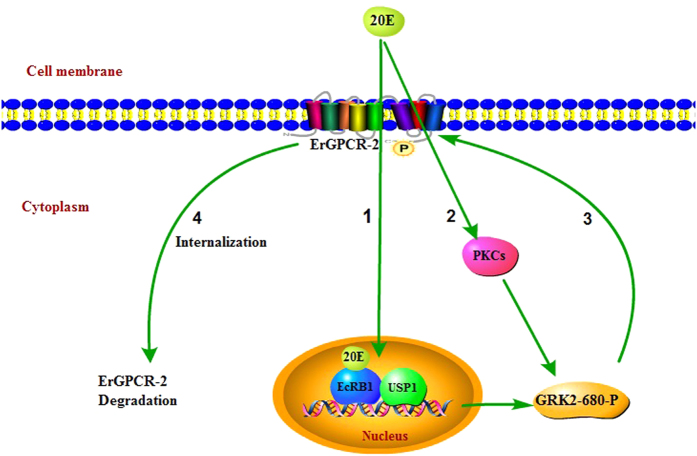
Diagram interpreting the function and mechanism of GRK2 in the 20E signal pathway. (1) 20E up-regulates the expression of *GRK2* by EcR-B1 and USP1; (2) 20E regulates PKC phosphorylation of GRK2 at serine 680 via ErGPCR-2; (3) The phosphorylated GRK2 moves toward the cell membrane, where it interacts with and phosphorylates ErGPCR-2; (4) The phosphorylated ErGPCR-2 is internalized to the cytoplasm to terminate 20E signaling^8^. Pathway Builder Tool 2.0 was used to draw the figure.

20E regulates PKC phosphorylation of GRK2 at serine 680 via ErGPCR-2. The phosphorylated GRK2 is transferred to the cell membrane to terminate the 20E signal (Fig. 7).

## Materials and Methods

### Animals

*H. armigera* larvae were raised on an artificial diet composed of powders from wheat germ and soybean with various vitamins and inorganic salts at 28 °C at 60–70% relative humidity and under light/dark cycles of 14:10 h in an insectarium^44^.

### *H. armigera* epidermal cell line culture

The *H. armigera* epidermal cell line (HaEpi) was established from the penultimate (5th) instar larval integument in our laboratory^45^. The cells were cultured as a loosely attached monolayer and maintained at 27 °C in tissue culture flasks. The tissue culture flasks had an area of 25 cm^2^ with 2 mL of antibiotic-free Grace’s medium supplemented with 10% heat-inactivated fetal bovine serum (FBS). The cells were subcultured weekly to a nearly confluent monolayer.

### Bioinformatic analysis of GRK2

Protein translation and predictions were analyzed using the ExPASy Proteomics Server (http://www.expasy.ch/tools/). Protein domain predictions were performed using SMART (http://smart.embl-heidelberg.de/). A phylogenetic tree was produced using the neighbor-joining method in MEGA 3.1 (http://www.megasoftware.net/) (Figures S1, 2 and 3).

### Recombinant expression and polyclonal antibody preparation

An 88-amino-acid fragment of GRK2 (aa30~aa118) was expressed in *Escherichia coli* Rosetta host cells as inclusion bodies using the vector PET30a with *GRK2*-EcoRI-F and *GRK2*-XhoI-R primers. The inclusion proteins were separated by SDS-PAGE. The antiserum was prepared using a previously described method^46^. The specificity of the antiserum was determined by western blot analysis and the antiserum was used for experiments without purification (Figure S4).

### Quantitative real-time reverse transcription PCR

Total larval RNA was reverse-transcribed from first-strand cDNA as the template for qRT-PCR using the primers listed in Table 1. The 10 μL mixture, which was used to perform qRT-PCR, consisted of 5 μL of SsoFast^TM^ EvaGreen Supermix (BioRad, California, USA), 1 μL of cDNA (diluted 1:10), 2 μL of 1 μM forward primers and 2 μL of 1 μM reverse primers. β-actin was used for control. Data were analyzed using the formula: *R* = 2^−[Δ*Ct* sample − Δ*Ct* control]^, where *R* is the relative expression level, Δ*Ct* sample is the difference between the *Ct* of the gene and the average β-actin in the experimental sample, and Δ*Ct* control is the difference between the Ct of the gene and the average of β-actin in the control sample.

### Hormone treatment in larvae

The 20E and JH III (Sigma, St. Louis, MO, USA) solutions were diluted to 0.1 mg/mL with phosphate-buffered saline (PBS, 10 mM Na_2_HPO_4_, 1.8 mM KH_2_PO_4_, 140 mM NaCl and 2.7 mM KCl, pH 7.4). 20E or JH III was injected in the sixth instar 6 h larvae (500 ng/larva). The untreated larvae were injected with an equivalent amount of DMSO dissolved in PBS.

### Western blot analysis

We used anti-β-actin to perform protein quantification^47^. The proteins were separated by SDS-PAGE and then transferred onto a nitrocellulose membrane. After blocking with 2% non-fat milk in TBS for 1 h at room temperature, the membrane was incubated with the antibody for 12 h. The membrane was washed three times with TBST (0.1% Tween-20 in TBS) for 15 min. Subsequently, the blot was probed with HRP-conjugated goat-anti-rabbit IgG (1:10000 in the blocking solution). 4-Chloro-1-naphthol was used as the HRP substrate to visualize the peroxidase activity.

### RNAi in the HaEpi cell line and larvae

A MEGA-script RNAi Kit (Ambion Inc, Austin, USA) was used to synthesize double-stranded RNA (dsRNA) with the PCR primers (*GRK2*-RNAi-F and *GRK2*-RNAi-R). Approximately 1 μg of *dsRNA* and 125 μL of FBS-free Grace’s medium with 8 μL of lipofectamine were mixed and added to the cells in 1 mL FBS-free Grace’s medium and incubated for 12 h. Subsequently, the cells were cultured with Grace’s medium with FBS for 12 h and incubated with 20E or JH III for 6 h. For the RNAi in larvae, *dsRNA* was diluted to 100 ng/μL. Approximately 5 μL of *dsRNA* was injected in a sixth instar-6 h larva hemoceol. The control larvae were injected individually with 5 μL of *dsGFP*. The *dsRNA* is cut to 21-23 nt siRNA in RNA interference^48^. In mammals, two or more 21-23 nt siRNA from different sequences are used to control the off target because long length *dsRNA* causes interference^49^. However, in insect, long length *dsRNA* can be used because it will produce multiple siRNA *in vitro* in theory. We used 540 bp *dsGRK2* for experiment. Experiments were performed in 30 larvae and repeated three times for student *t* test.

### Immunohistochemistry

The overexpression plasmid construction method: GRK2-GFP-His (ORF of GRK2), GRK2^T672A^-GFP-His (replacing threonine 672 with alanine) and GRK2^S680A^-GFP-His (replacing serine 680 with alanine) were overexpressed using the GFP-pIEx-His vector in HaEpi cells. The GPCR inhibitor suramin (sodium salt, final concentration, 50 μM), GDPβs (100 μg/mL) and PKC inhibitor CC (5 μM) (all from Sigma-Aldrich)^28^ were added to the cells for 60 minutes. Then, HaEpi cells were treated with 20E (1 μM) for 5 minutes. The cells were fixed in 4% paraformaldehyde for 30 min at room temperature. After washing with Dulbecco’s phosphate-buffered saline (DPBS: 8.10 mM Na_2_HPO_4_, 1.47 mM KH_2_PO_4_, 138 mM NaCl, and 2.67 mM KCl, pH 7.4), the cells were incubated with anti-GRK2 polyclonal antibody (1:500 ratio, diluted in blocking buffer) at 4 °C overnight. The cells were washed with DPBS and then incubated with the secondary antibody goat anti-rabbit-Alexa Fluor 488 (1:1,000 ratio, diluted in blocking buffer) at 37 °C for 1 h. The cells were then stained at the cell membrane using wheat germ agglutinin (WGA, red) for 15 min at room temperature. Pre-serum was used as the negative control. Fluorescence was observed using a Zeiss LSM 700 laser confocal microscope (Zeiss, Thornwood, NY).

### Co-immunoprecipitation

HaEpi cells were pre-treated with 20E for 30 min; DMSO as a control. The proteins were extracted from the cells with radioimmunoprecipitation assay (RIPA) buffer (0.1 M Tris–HCl buffer, pH 8.0 containing 0.15 M NaCl, and 1% NP-40) and harvested by centrifugation at 12,000 × *g* for 20 min at 4 °C. Approximately 30 μL to 40 μL of supernatant was used as the input protein. The remainder of the supernatant was added to the antiserum against GRK2, incubated for 3 h with gentle shaking at 4 °C, and then incubated with Protein A resin for 3 h with gentle shaking at 4 °C. After centrifugation at 12,000 × *g* for 10 min at 4 °C, the supernatant was discarded. After washing with PBS, the resin was treated with SDS-PAGE loading buffer and boiled for 10 min for western blot analysis with various antibodies.

### Isolation of cell membrane and cytoplasmic proteins

A membrane and cytoplasmic protein extraction kit (Beyotime, Haimen, China) was used to separate the membrane and cytoplasmic proteins according to the manufacturer’s instructions.

### NucView^TM^ 488 Caspase-3 Assay

One μg of *dsGRK2* and 125 μL of physiological saline medium with 8 μL of Transfection reagents (Biotium, Inc., Hayward, CA United States) were mixed and added to the cells in 2 mL of Grace’s medium and then incubated for 24 h. Subsequently, the cells were incubated with 20E (1 μM) for 72 h. The control was incubated with *dsGFP*. A NucView^TM^ 488 Caspase-3 Assay Kit for live cells (Biotium, USA) was used to measure caspase-3 activity^50^. Grace’s medium was removed and replaced with DPBS containing 5 μM NucView^TM^ 488 Caspase-3 substrate stock solution, and the cells were incubated with the substrate for 30 min at room temperature.

### GRK2 phosphorylation levels detection

We overexpressed GRK2-GFP-His, GRK2^T672A^-GFP-His or GRK2^S680A^-GFP-His respectively in cells. The cells were incubated with 20E (1 μM) for 5 min. DMSO was used as negative control. GRK2-GFP-His, GRK2^T672A^-GFP- His or GRK2^S680A^-GFP-His was purified by Ni^2+^-NTA affinity column for detecting phosphorylation levels. We used the Phosphoprotein phosphate estimation assay kit (Sangon Biotech, Shanghai, China) to detect the number of moles of phosphorus per mole of GRK2-GFP-His, GRK2^T672A^-GFP-His or GRK2^S680A^-GFP-His based on the alkaline hydrolysis of phosphate from seryl and threonyl residues in phosphoproteins.

### Acceptor photobleaching fluorescence resonance energy transfer (Ap-Fret)

Cells overexpressed ErGPCR-2-CFP-His, GRK2-YFP-His and ErGPCR-2-CFP-His+GRK2-YFP-His for 48 h. The cyan fluorescent protein (CFP) and yellow fluorescent protein (YFP) were cloned from a CMV-Brainbow-1.1 M plasmid (Addgene). The cells were incubated with 20E (1 μM) for 10 min, and DMSO was used as a solvent control. The cells were fixed with 4% paraformaldehyde for 30 min and then washed with DPBS. A Zeiss LSM 780 laser scanning system was used to observe the results. CFP was excited using a 458 nm Argon/2 laser, and the emission was detected using a 465–510 nm filter; YFP was excited using a 514 nm Argon/2 laser, and the emission was detected using a 520–555 nm filter. Images were acquired by exciting at 488 and 514 nm to confirm the original expression of ErGPCR-2-CFP and GRK2-YFP, respectively, prior to acceptor photobleaching. Images for ErGPCR-2-CFP were collected before and after photobleaching with the 514 nm Argon/2 laser at the maximum intensity. The signal intensity of CFP was measured with ImageJ image software, and the FRET efficiency was calculated using the following formula: FRET efficiency (%) = (I_post_ − I_pre_) × 100/I_post_, where I_pre_ and I_post_ correspond to the CFP signal intensities (background signal was subtracted) before and after photobleaching, respectively.

## Additional Information

**How to cite this article**: Zhao, W.-L. *et al*. G-protein-coupled receptor kinase 2 terminates G-protein-coupled receptor function in steroid hormone 20-hydroxyecdysone signaling. *Sci. Rep*. **6**, 29205; doi: 10.1038/srep29205 (2016).

## Supplementary Material

Supplementary Information

## Figures and Tables

**Table 1:** Primers used in PCR.

Primer name	(5′ → 3′) nucleotide sequence
GRK2-F1- EcoRI	tactcagaattcaaaaagattgttctgcca
GRK2-R1-Xho I	tactcactcgagtcagccgatgatgcggtg
GRK2-RNAi-F	gcgtaatacgactcactatagggcgactttagcgtgcaccg
GRK2-RNAi-R	gcgtaatacgactcactatagggtagcccgtgccttttgac
GFP-RNAi-F	gcgtaatacgactcactataggtggtcccaattctcgtggaac
GFP-RNAi-R	gcgtaatacgactcactataggagctggagacaactcctcacg
GRK2-G/YFP-F	tactcacaattggatggcggacctcgaggct
GRK2-G/YFP-R	tactcaggcgcgccgagttgctcccgttgggcgc
GRK2-T672A-GFP-F	atctacggcgcggacggcgccaa
GRK2-T672A-GFP-R	ttggcgccgtccgcgccgtagat
GRK2-S680A-GFP-F	aaggacgccatggcgctggtgcg
GRK2-S680A-GFP-R	cgcaccagcgccatggcgtcctt
GPCR-2-C/YFP-F	tactcagagctcatgattacattcataacagtg
GPCR-2-C/YFP-R	tactcacaattgggaggctgtttgatgttgagcga
GRK2-QRT-F	aaagtgacaaatacacgaggtt
GRK2-QRT-F	cttggacctgatgaacgg
Actin-QRT-F	cctggtattctgaccgtatgc
Actin-QRT-R	ctgttggaaggtggagagggaa
EcR-B1-QRT-F	aattgcccgtcagtacga
EcR-B1-QRT-R	tgagcttctcattgagga
USP1-QRT-F	ggtcctgacagcaatgtt
USP1-QRT-R	ttccagctccagctgactgaag
HR3-QRT-F	tcaagcacctcaacagcagcccta
HR3-QRT-R	gactttgctgatgtcaccctccgc
Br-QRT-F	ggtgactgtccttactgcggcat
Br-QRT-R	ttaattcctttgaccatgact
Kr-h1-F	gcggtgggcctccacgtgtcgaacg
Kr-h1-R	cgttcgacacgtggaggcccaccgc

## References

[b1] Marinissen M. J. & Gutkind J. S. G-protein-coupled receptors and signaling networks: emerging paradigms. Trends Pharmacol. Sci. 22, 368–76 (2001). 11431032 10.1016/s0165-6147(00)01678-3

[b2] Belmonte S. L. & Blaxall B. C. G Protein coupled receptor kinases as therapeutic targets in cardiovascular disease. Circ.Res. 109, 309–319 (2011). 21778435 10.1161/CIRCRESAHA.110.231233 PMC3146028

[b3] Seachrist J. L. & Ferguson S. S. Regulation of G protein-coupled receptor endocytosis and trafficking by Rab GTPases. Life Sci. 74, 225–235 (2003). 14607250 10.1016/j.lfs.2003.09.009

[b4] Zhang X.-Q. . β-Arrestin1 interacts with G protein-coupled receptor to desensitize signaling of the steroid hormone 20-hydroxyecdysone in the lepidopteran insect *Helicoverpa armigera*. Cell Signal 27, 878–886 (2015). 25660147 10.1016/j.cellsig.2015.01.016

[b5] Revankar C. M., Cimino D. F., Sklar L. A., Arterburn J. B. & Prossnitz E. R. A transmembrane intracellular estrogen receptor mediates rapid cell signaling. Science 307, 1625–30 (2005). 15705806 10.1126/science.1106943

[b6] Manaboon M., Iga M., Iwami M. & Sakurai S. Intracellular mobilization of Ca^2+^ by the insect steroid hormone 20-hydroxyecdysone during programmed cell death in silkworm anterior silk glands. J. Insect. Physiol. 55, 123–129 (2009). 10.1016/j.jinsphys.2008.10.013 19041319

[b7] Cai M.-J. . G-protein-coupled receptor participates in 20-hydroxyecdysone signaling on the plasma membrane. Cell. Commun. Signal. 12, 9, doi: 10.1186/1478-811X-12-9 (2014). 24507557 PMC3937218

[b8] Wang D., Zhao W.-L., Cai M.-J., Wang J.-X. & Zhao X.-F. G-protein-coupled receptor controls steroid hormone signaling in cell membrane. Sci. Rep-UK. 5, 8675, doi: 10.1038/srep08675 (2015). PMC4345324 25728569

[b9] Homan K. T., Glukhova A. & Tesmer J. J. Regulation of G protein-coupled receptor kinases by phospholipids. Curr. Med. Chem. 20, 39–46 (2013). 23151001

[b10] Inglese J., Glickman J., Lorenz W., Caron M. & Lefkowitz R. Isoprenylation of a protein kinase. Requirement of farnesylation/alpha-carboxyl methylation for full enzymatic activity of rhodopsin kinase. J. Biol. Chem. 267, 1422–1425 (1992). 1730692

[b11] Pitcher J. A. . Role of beta gamma subunits of G proteins in targeting the beta-adrenergic receptor kinase to membrane-bound receptors. Science 257, 1264–1267 (1992). 1325672 10.1126/science.1325672

[b12] Boekhoff I. . Olfactory desensitization requires membrane targeting of receptor kinase mediated by beta gamma-subunits of heterotrimeric G proteins. J. Biol. Chem. 269, 37–40 (1994). 8276821

[b13] Stoffel R. H., Inglese J., Macrae A. D., Lefkowitz R. J. & Premont R. T. Palmitoylation increases the kinase activity of the G protein-coupled receptor kinase, GRK6. Biochemistry 37, 16053–16059 (1998). 9819198 10.1021/bi981432d

[b14] Pitcher J. A. . Phosphatidylinositol 4, 5-bisphosphate (PIP2)-enhanced G protein-coupled receptor kinase (GRK) activity: Location, structure, and regulation of the PIP2 binding site distinguishes the GRK subfamilies. J. Biol. Chem. 271, 24907–24913 (1996). 8798768 10.1074/jbc.271.40.24907

[b15] Cong M. . Regulation of membrane targeting of the G protein-coupled receptor kinase 2 by protein kinase A and its anchoring protein AKAP79. J. Biol. Chem. 276, 15192–15199 (2001). 11278469 10.1074/jbc.M009130200

[b16] Winstel R., Freund S., Krasel C., Hoppe E. & Lohse M. J. Protein kinase cross-talk: membrane targeting of the beta-adrenergic receptor kinase by protein kinase C. P. Natl. Acad. Sci. USA 93, 2105–2109 (1996). 10.1073/pnas.93.5.2105 PMC39917 8700892

[b17] Chuang T. T., LeVine H. & De Blasi A. Phosphorylation and activation of β-adrenergic receptor kinase by protein kinase C. J. Biol. Chem. 270, 18660–18665 (1995). 7629197 10.1074/jbc.270.31.18660

[b18] Pals-Rylaarsdam R., Xu Y., Witt-Enderby P., Benovic J. L. & Hosey M. M. Desensitization and internalization of the m2 muscarinic acetylcholine receptor are directed by independent mechanisms. J. Biol. Chem. 270, 29004–29011 (1995). 7499433 10.1074/jbc.270.48.29004

[b19] Bruch R. C., Kang J., Moore M. L. & Medler K. F. Protein kinase C and receptor kinase gene expression in olfactory receptor neurons. J. Neurobiol. 33, 387–394 (1997). 9322156 10.1002/(sici)1097-4695(199710)33:4<387::aid-neu4>3.0.co;2-6

[b20] Peppel K. . G protein-coupled receptor kinase 3 (GRK3) gene disruption leads to loss of odorant receptor desensitization. J. Biol. Chem. 272, 25425–25428 (1997). 9325250 10.1074/jbc.272.41.25425

[b21] Liu J., Shi G. P., Zhang W. Q., Zhang G. R. & Xu W. H. Cathepsin L function in insect moulting: molecular cloning and functional analysis in cotton bollworm, Helicoverpa armigera. Insect. Mol. Biol. 15, 823–834 (2006). 17201774 10.1111/j.1365-2583.2006.00686.x

[b22] Liu C.-Y., Zhao W.-L., Wang J.-X. & Zhao X.-F. Cyclin-dependent kinase regulatory subunit 1 promotes cell proliferation by insulin regulation. Cell Cycle 14, 3045–3057 (2015). 26199131 10.1080/15384101.2015.1053664 PMC4825559

[b23] Terenius O. . RNA interference in Lepidoptera: an overview of successful and unsuccessful studies and implications for experimental design. J. Insect Physiol. 57, 231–245 (2011). 21078327 10.1016/j.jinsphys.2010.11.006

[b24] Abbracchio M. P. . International Union of Pharmacology LVIII: update on the P2Y G protein-coupled nucleotide receptors: from molecular mechanisms and pathophysiology to therapy. Pharmacol. Rev. 58, 281–341 (2006). 16968944 10.1124/pr.58.3.3 PMC3471216

[b25] Khakh B. S. . International union of pharmacology. XXIV. Current status of the nomenclature and properties of P2X receptors and their subunits. Pharmacol. Rev. 53, 107–118 (2001). 11171941

[b26] Wolner I., Kassack M. U., Ullmann H., Karel A. & Hohenegger M. Use-dependent inhibition of the skeletal muscle ryanodine receptor by the suramin analogue NF676. Brit. J. Pharmacol. 146, 525–533 (2005). 16056233 10.1038/sj.bjp.0706359 PMC1751178

[b27] Beindl W. . Inhibition of receptor/G protein coupling by suramin analogues. Mol. Pharmacol. 50, 415–423 (1996). 8700151

[b28] Chmura S. J. . *In vitro* and *in vivo* activity of protein kinase C inhibitor chelerythrine chloride induces tumor cell toxicity and growth delay *in vivo*. Clin. Cancer. Res. 6, 737–742 (2000). 10690561

[b29] Dominguez R., Hu E., Zhou M. & Baudry M. 17β-estradiol-mediated neuroprotection and ERK activation require a pertussis toxin-sensitive mechanism involving GRK2 and β-arrestin-1. J. Neurosci. 29, 4228–4238 (2009). 19339617 10.1523/JNEUROSCI.0550-09.2009 PMC3182118

[b30] Ansonoff M. A. & Etgen A. M. Estrogen increases G protein coupled receptor kinase 2 in the cortex of female rats. Brain. Res. 898, 186–9 (2001). 11292465 10.1016/s0006-8993(01)02161-8

[b31] Mak J. C., Hisada T., Salmon M., Barnes P. J. & Chung K. F. Glucocorticoids reverse IL‐1β‐induced impairment of β-adrenoceptor-mediated relaxation and up-regulation of G-protein-coupled receptor kinases. Brit. J. Pharmacol. 135, 987–996 (2002). 11861327 10.1038/sj.bjp.0704545 PMC1573209

[b32] Riddiford L. M., Hiruma K., Zhou X. & Nelson C. A. Insights into the molecular basis of the hormonal control of molting and metamorphosis from *Manduca sexta* and *Drosophila melanogaster*. Insect. Biochem. Molec. 33, 1327–1338 (2003). 10.1016/j.ibmb.2003.06.001 14599504

[b33] Kumar D. . Endogenous 20-hydroxyecdysone levels in the haemolymph of non-diapause-destined and diapause-destined generations of tasar silkworm, *Antheraea mylitta* (Lepidoptera: Saturniidae) and associated developmental changes. Eur. J. Entomol. 105, 591–598 (2008).

[b34] Kunapuli P., Gurevich V. V. & Benovic J. L. Phospholipid-stimulated autophosphorylation activates the G protein-coupled receptor kinase GRK5. J. Biol. Chem. 269, 10209–10212 (1994). 8144599

[b35] Elorza A., Sarnago S. & Mayor F. Agonist-dependent modulation of G protein-coupled receptor kinase 2 by mitogen-activated protein kinases. Mol. Pharmacol. 57, 778–783 (2000). 10727525 10.1124/mol.57.4.778

[b36] Penela P., Elorza A., Sarnago S. & Mayor F. β-arrestin and c-Src-dependent degradation of G-protein-coupled receptor kinase 2. EMBO. J. 20, 5129–5138 (2001). 11566877 10.1093/emboj/20.18.5129 PMC125273

[b37] Koch W. J., Inglese J., Stone W. & Lefkowitz R. The binding site for the beta gamma subunits of heterotrimeric G proteins on the beta-adrenergic receptor kinase. J. Biol. Chem. 268, 8256–8260 (1993). 8463335

[b38] Liu Z. . TLR4 Signaling augments monocyte chemotaxis by regulating G protein-coupled receptor kinase 2 translocation. J. Immunol. 191, 857–864 (2013). 23772028 10.4049/jimmunol.1300790 PMC3702632

[b39] Dale L. B. . G protein-coupled receptor kinase-mediated desensitization of metabotropic glutamate receptor 1A protects against cell death. J. Biol. Chem. 275, 38213–38220 (2000). 10982802 10.1074/jbc.M006075200

[b40] Ni Y., Sinnett-Smith J., Young S. H. & Rozengurt E. PKD1 mediates negative feedback of PI3K/Akt activation in response to G protein-coupled receptors. PloS one 8, e73149 (2013). 24039875 10.1371/journal.pone.0073149 PMC3767810

[b41] Gidon A. . Endosomal GPCR signaling turned off by negative feedback actions of PKA and v-ATPase. Nat. Chem. Biol. 10, 707–709 (2014). 25064832 10.1038/nchembio.1589 PMC4138287

[b42] Carman C. V. & Benovic J. L. G-protein-coupled receptors: turn-ons and turn-offs. Curr. Opin. Neurobiol. 8, 335–344 (1998). 9687355 10.1016/s0959-4388(98)80058-5

[b43] Gurevich V. V. . Arrestin Interactions with G Protein-coupled Receptors direct binding studies of wild type and mutant arrestins with rhodopsin, β2-adrenergic, and m2 muscarinic cholinergic receptors. J. Biol. Chem. 270, 720–731 (1995). 7822302 10.1074/jbc.270.2.720

[b44] Zhao X.-F., Wang J.-X. & Wang Y.-C. Purification and characterization of a cysteine proteinase from eggs of the cotton boll worm, *Helicoverpa armigera*. Insect. Biochem. Molec. 28, 259–264 (1998).

[b45] Shao H.-L. . Establishment of a new cell line from lepidopteran epidermis and hormonal regulation on the genes. PLoS One 3, e3127 (2008). 18769621 10.1371/journal.pone.0003127 PMC2518862

[b46] Dong D.-J., Liu W., Cai M.-J., Wang J.-X. & Zhao X.-F. Steroid hormone 20-hydroxyecdysone regulation of the very-high-density lipoprotein (VHDL) receptor phosphorylation for VHDL uptake. Insect. Biochem. Molec. 43, 328–335 (2013). 10.1016/j.ibmb.2013.02.001 23416133

[b47] de Kok J. B. . Normalization of gene expression measurements in tumor tissues: comparison of 13 endogenous control genes. Lab. Invest. 85, 154–159 (2005). 15543203 10.1038/labinvest.3700208

[b48] Fire A. . Potent and specific genetic interference by double-stranded RNA in *Caenorhabditis elegans*. Nature 391, 806–811 (1998). 9486653 10.1038/35888

[b49] Takahashi Y. . Persistent interferon transgene expression by RNA interference-mediated silencing of interferon receptors. J. Gene. Med. 12, 739–746 (2010). 20821744 10.1002/jgm.1493

[b50] Cen H., Mao F., Aronchik I., Fuentes R. J. & Firestone G. L. DEVD-NucView488: a novel class of enzyme substrates for real-time detection of caspase-3 activity in live cells. Faseb. J. 22, 2243–2252 (2008). 18263700 10.1096/fj.07-099234

