# Supplementary material for: G-protein-coupled receptor kinase 2 terminates G-protein-coupled receptor function in steroid hormone 20-hydroxyecdysone signaling
Source: Sci Rep. 2016 Jul 14;6:29205. doi: 10.1038/srep29205 (PMC4944123; doi:10.1038/srep29205)
Supplement: Supplementary Information [file srep29205-s1.pdf]

G-protein-coupled receptor kinase 2 terminates G-protein-coupled receptor function in steroid hormone 20-hydroxyecdysone signaling

Wen-Li Zhao, Di Wang, Chun-Yan Liu, Xiao-Fan Zhao *

Shandong Provincial Key Laboratory of Animal Cells and Developmental Biology, School of Life Sciences, Shandong University, Jinan, Shandong 250100, China

Running title: GRK2 terminates GPCR function in 20E pathway

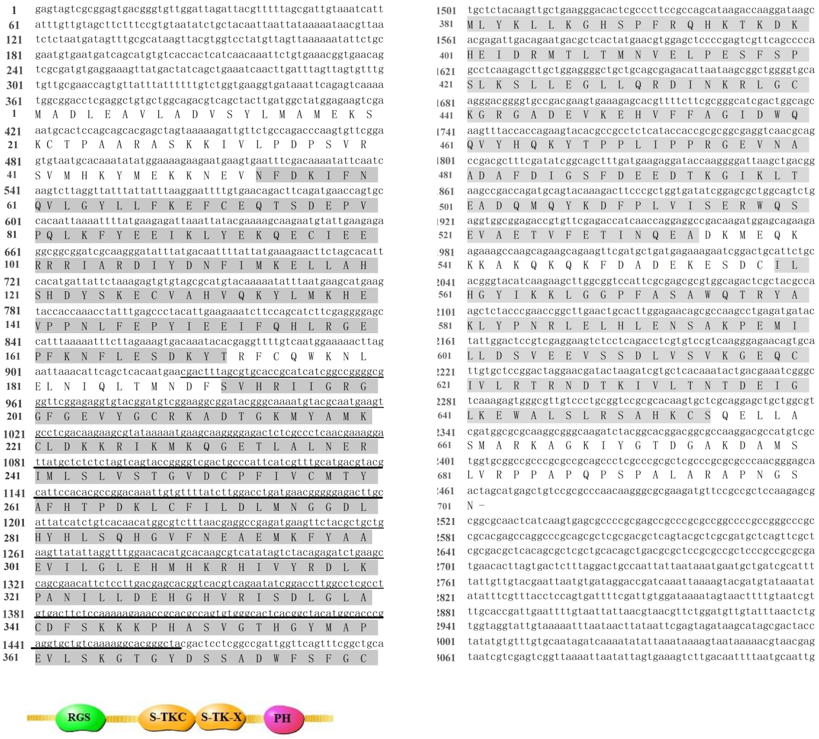

Fig. S1 Nucleotide and deduced amino acid sequence of *GRK2* from *H. amigera*. Gray sequence indicates the *GRK2* subfamily domain. Double-stranded RNA interference alignments are marked by underline. The ORF sequence of *GRK2* is 2103 bp, encodes 701 amino acid proteins with molecular mass 78 kDa. GRK2 has four structural domains of RGS, S-TKC, S-TK-X and PH.

* **Corresponding author**: Xiao-Fan Zhao, **Tel and Fax**: 86-531-88364620. **E-mail**: xfzhao@sdu.edu.cn

The nucleotide sequence of GRK2 has been submitted to GenBank with accession number KT364485. The sequence will be released in Dec 30, 2016 in GenBank.

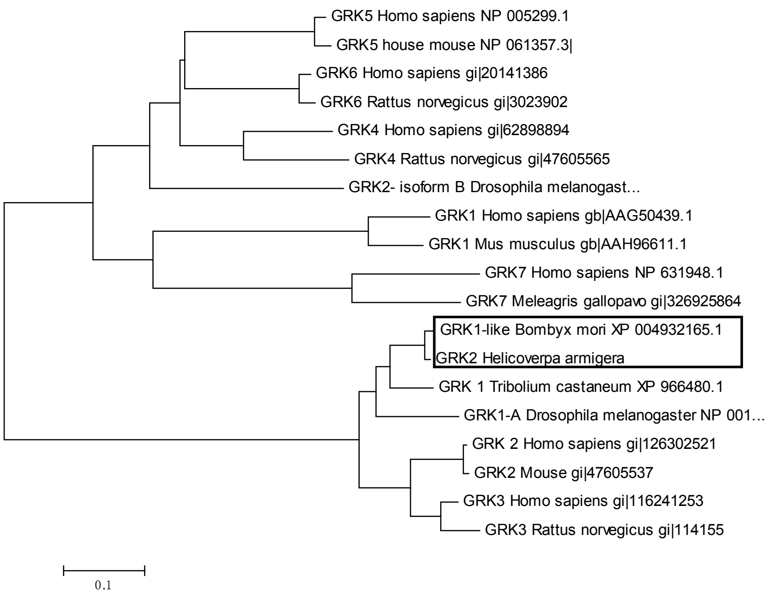

Fig. S2 Phylogenetic analysis of *GRK2* with similar proteins from other species was conducted using MEGA 4.0. GRK2 of *H. armigera* shares most similarity to GRK1-like of *Bombyx mori.*

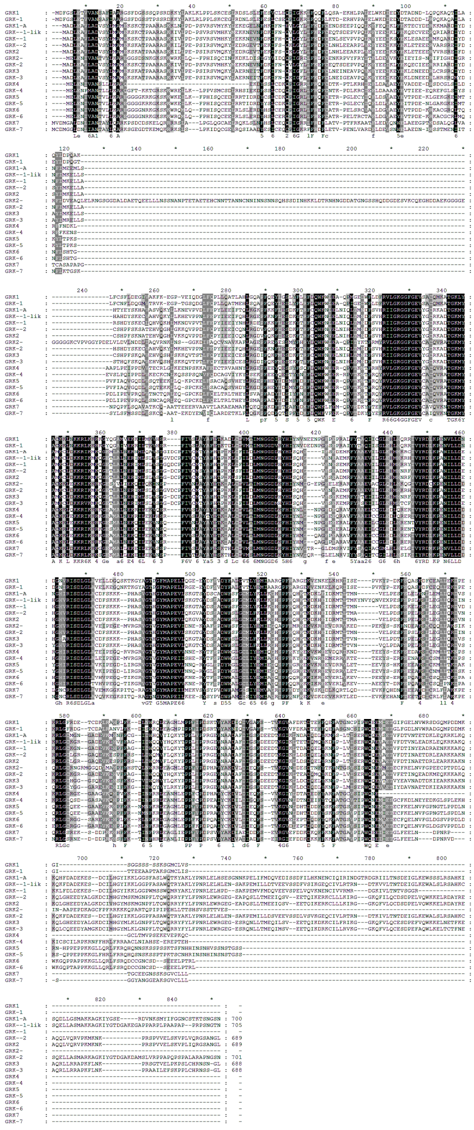

Fig. S3 Alignment of *Helicoverpa armigera* GRK2 with other GRK proteins from other species. The conserve amino acids are in dark. The Genbank numbers are same as Fig. S2.

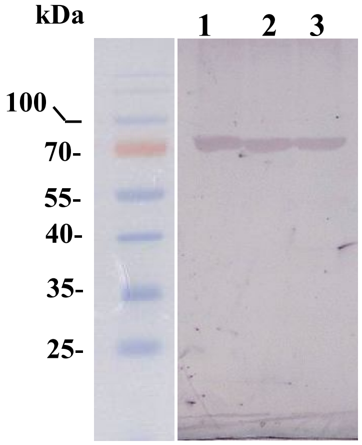

Figure S4. The specificity of rabbit polyclonal antibody of GRK2. Western blots to detect the specificity of the antiserum with the 6th instar 48 h integument proteins (lane 1), 6-48 h midgut proteins (lane 2) and 6-48 h fat body proteins (lane 3).

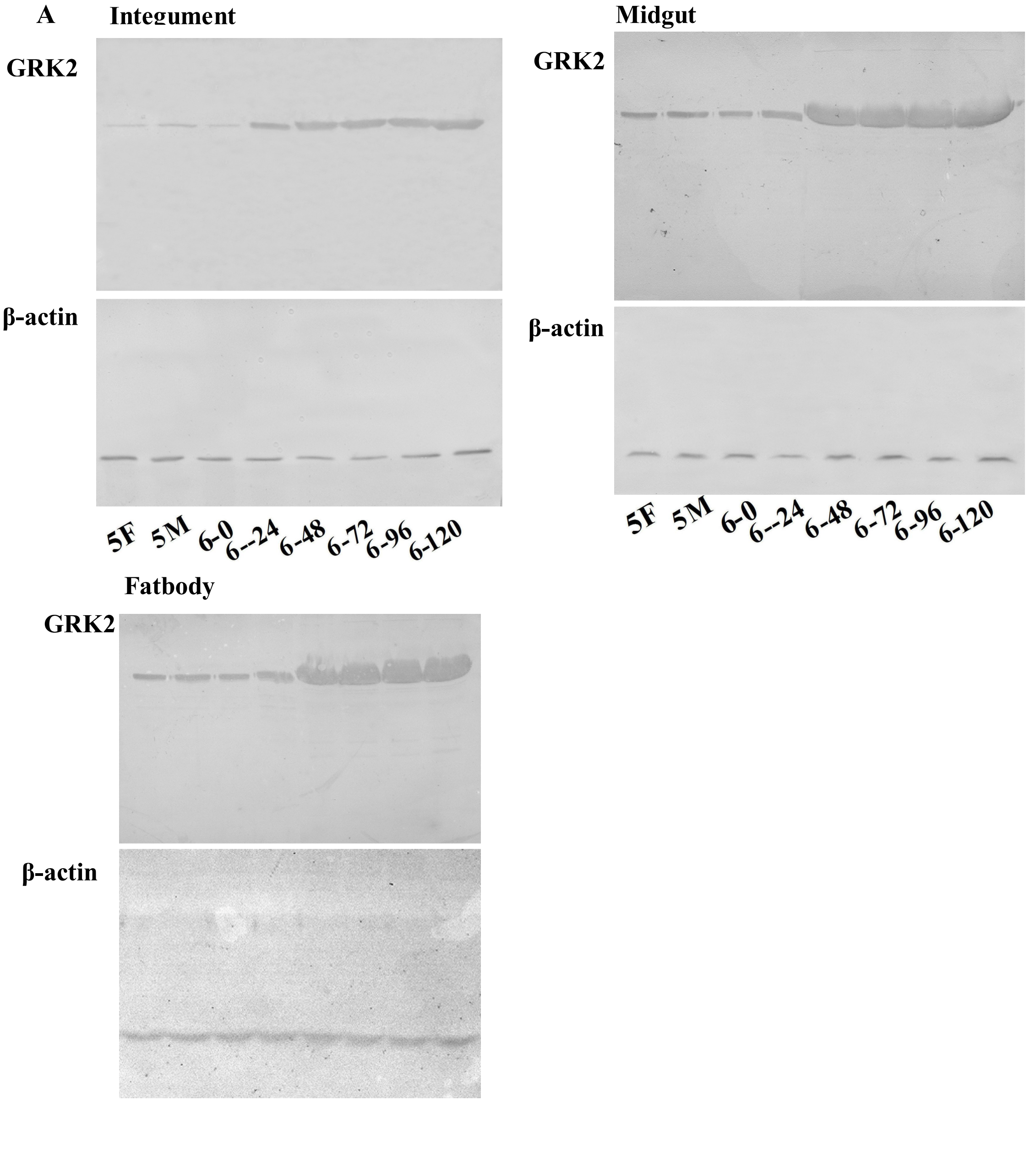

B

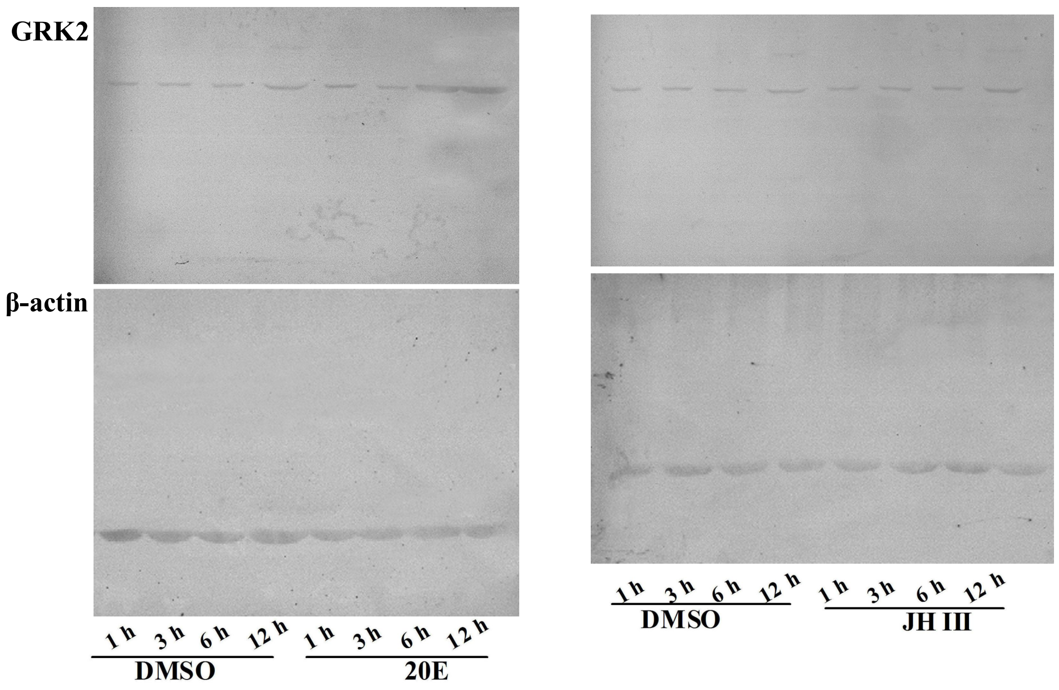

Figure S5. Full-length blots data of Figure 1A and B

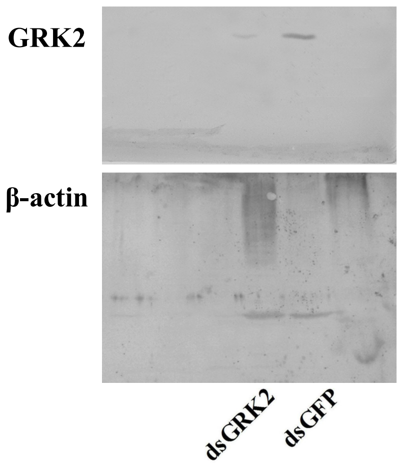

Figure S6. Full-length blots data of Figure 2B

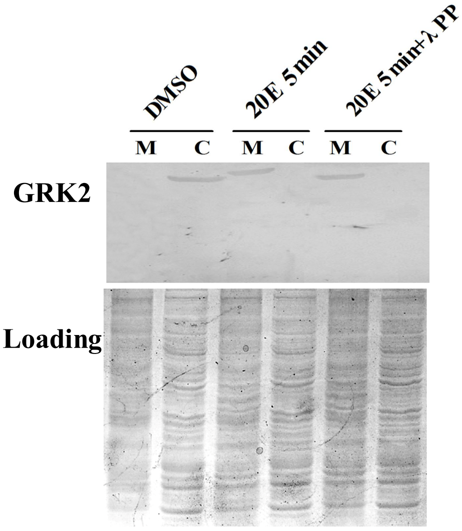

Figure S7. Full-length blots data of Figure 4C.

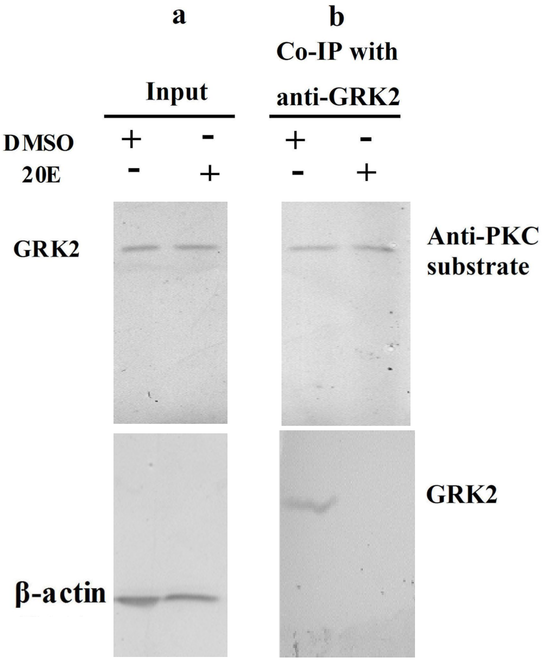

Figure S8. Full-length blots data of Figure 4D.

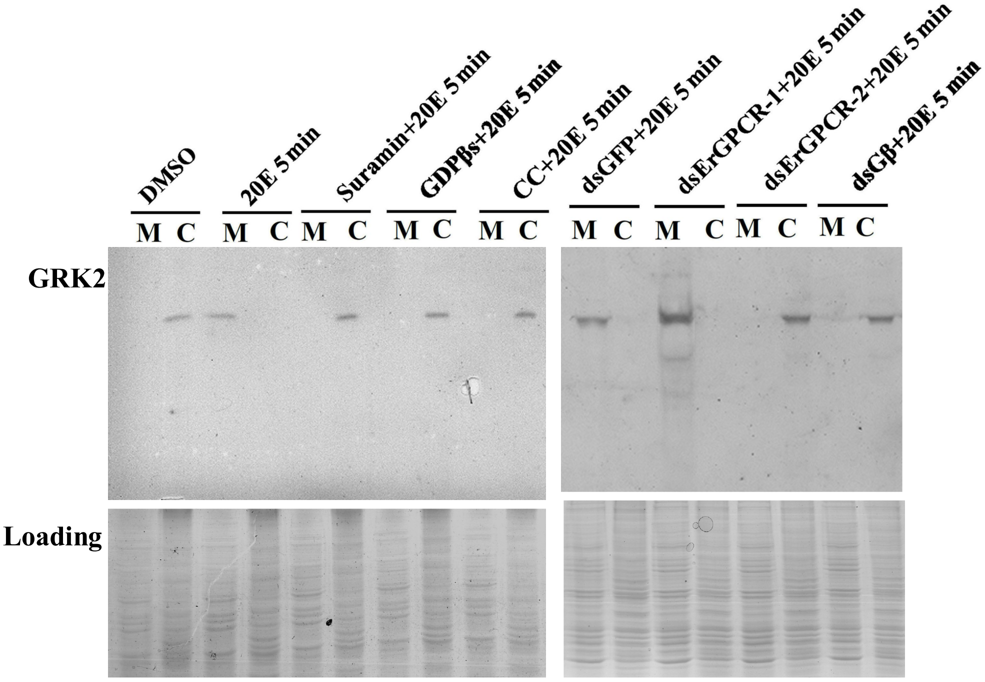

Figure S9. Full-length blots and gels data of Figure 4E.

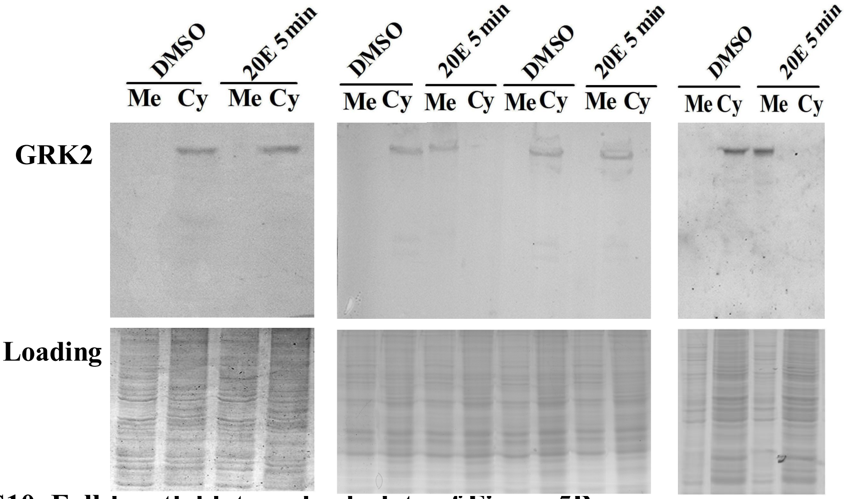

Figure S10. Full-length blots and gels data of Figure 5B.

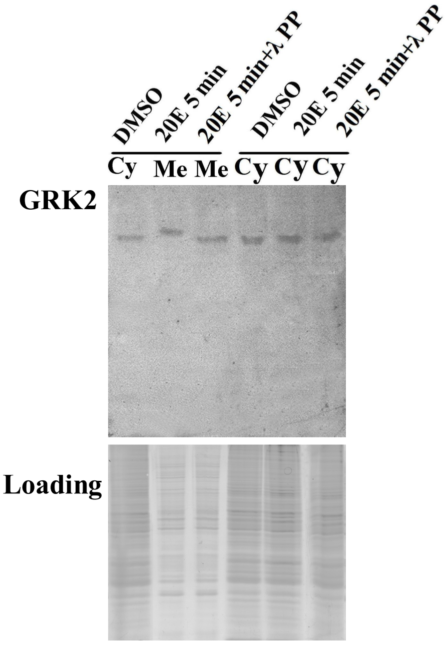

Figure S11. Full-length blots and gels data of Figure 5C.
